# Laparoscopic repair of a right para duodenal hernia

**DOI:** 10.4103/0972-9941.59313

**Published:** 2009

**Authors:** Vishnu Bhartia, Anil Kumar, Indira Khedkar, K S Savita, N Goel

**Affiliations:** Institute of Minimally Invasive Surgery (IMIS), A.M.R.I. Hospital, Dhakuria, Kolkata, India

**Keywords:** Laparoscopy, small bowel obstruction, para duodenal hernia

## Abstract

Para duodenal hernia is among the uncommon and rare causes of intestinal obstruction, but it is the most common type of internal hernia in abdomen and accounts for more than half of cases that do occur. Here, we are reporting a case of right Para duodenal hernia, reduced and repaired laparoscopically. This thirteen year old girl presented to us with features of small bowel obstruction of two days duration. Plain abdominal X-ray showed multiple fluid levels confined to right side of abdomen. A diagnostic laparoscopy was done under General Anaesthesia. Right Para duodenal hernia was found with small bowel confined to the right side between the ascending colon and hepatic flexure of colon. Laparoscopic reduction of contents of the hernia was done starting from the Ileocaecal junction. Hernial opening was closed laparoscopically with nonabsorbable suture. Patient is quite well till date and has had no recurrence of symptoms

## INTRODUCTION

Para duodenal hernias are among the uncommon and rare causes of intestinal obstruction and account for about 53% of cases of internal hernia. Diagnosis is often made perioperatively as clinical symptoms may be intermittent and nonspecific and include pain abdomen, nausea, vomiting and distension of abdomen.[1] About 75% occur on the left side (Fossa of Landzert) and remaining 25% on the right. CT scan abdomen has an important role in the diagnostic workup of Para duodenal hernia. Here, we are presenting a case of intestinal obstruction caused by right Para duodenal hernia reduced and repaired successfully by laparoscopy.

## CASE REPORT

**Figure 1:**
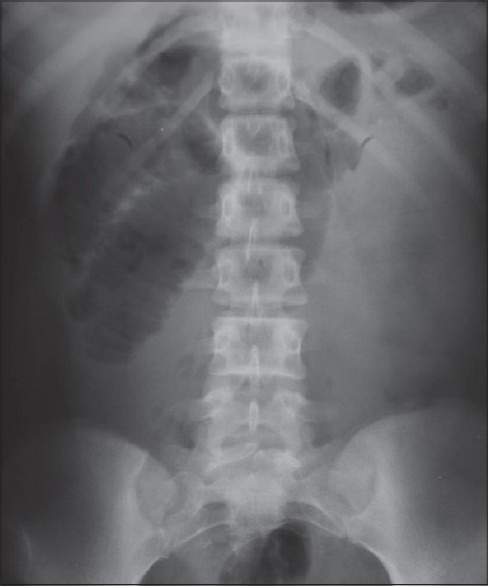
Distended bowel loops on the right side of the abdomen

**Figure 2:**
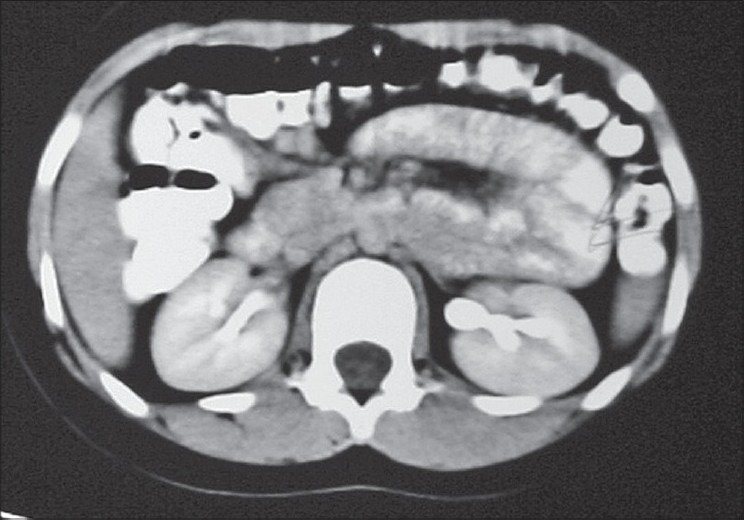
Axial CT, showing distended loop on the left side

This thirteen year old girl presented to us with features of small bowel obstruction of two days duration starting with pain abdomen, vomiting and abdominal distension. Abdomen was distended with mild tenderness and obstructive bowel sounds. Plain X-ray abdomen revealed multiple small bowel fluid levels mainly on the right side [Figure 1]. She had a past history of similar episode at nine years of age. CT scan of abdomen done at that time showed thickened loops of small bowel mainly in the left side of abdomen [Figure 2]. She had resolved spontaneously on conservative treatment. This time all her other clinical parameters including hematology, serum electrolytes, liver function test, chest X-ray, ECG were within normal limits.

**Figure 3:**
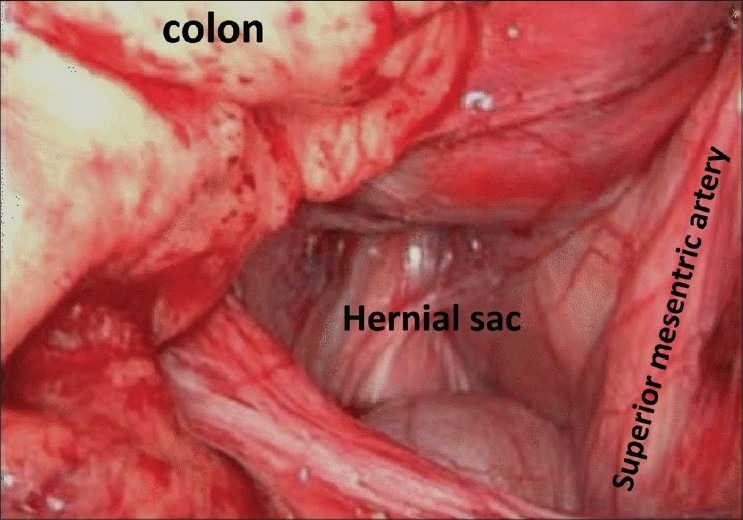
Laparoscopic view of the hernial sac and its boundary

**Figure 4:**
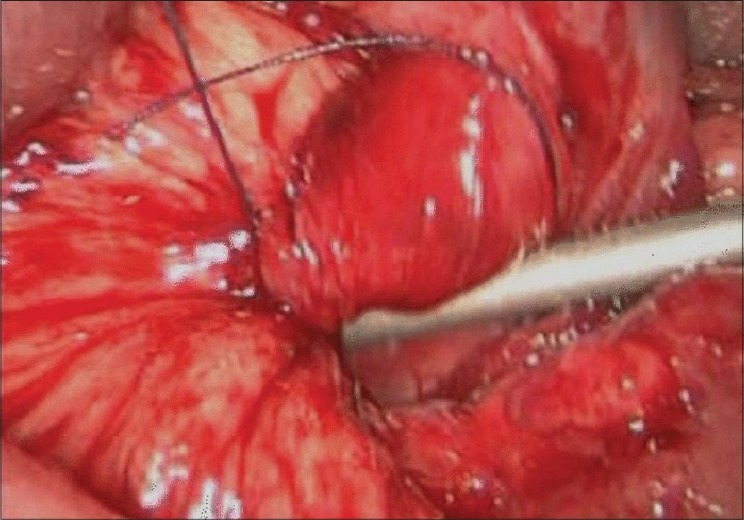
Laparoscopic closure of the hernial sac

A diagnostic laparoscopy was undertaken under General Anaesthesia. Four ports were inserted. Almost the whole small bowel was found distended and confined to the upper abdomen.Ileocaecal junction was identified. Collapsed loop was traced to an opening in the retroperitoneum. Rest of small bowel was gradually withdrawn into the peritoneal cavity. A few loops were dusky in color but all were viable. On reducing the whole small bowel, it was observed that it was a large right Para duodenal hernia [Figure 3]. Hernial opening was closed laparoscopically with nonabsorbable running suture [Figure 4].

Postoperatively, she developed ileus. Contrast CT scan was done on third day which showed one thickened small bowel loop on left side but without any obstruction. Thereafter, she recovered gradually and was discharged on the fifth postoperative day She remains well till date and had no recurrence of abdominal symptoms.

## DISCUSSION

Paraduodenal hernias are rare congenital anomalies of midgut malrotation[1,2] with less than 500 cases reported in the world's literature. It accounts for about 53% of all internal hernias but only 0.2-0.9% of all cases of small bowel obstructions.[2] Exact cause is obscure but opinion is that it is the result of malrotational abnormalities of midgut and failure of mesentery to fuse with parietal peritoneum.[3] Paraduodenal hernias are classified into left sided or right sided based on relationship of mesenteric vessels to the small bowel. Left sided Para duodenal hernia is more common (75%) than right sided with a slight male preponderance.[4]

Left sided Para duodenal hernias develop through a peritoneal defect to the left of fourth part of duodenum (fossa of Landzert); right margin (mouth) of the hernial sac is formed by inferior mesenteric vein. Right sided paraduodenal hernia is due to midgut malrotation and failure of mesentery to fuse with the parietal peritoneum causing hernial defect and protrusion of small bowel through mesentricoparietal fossa, as in our case. Superior mesenteric artery forms the left margin of the hernial sac.[2]

In abdominal radiograph, both types of paraduodenal hernia show displaced small bowel loops confined in a sac on one side of the midline with features of small bowel obstruction.

CT scan of abdomen plays an important role in the diagnosis of paraduodenal hernia.[5] Most commonly seen signs of paraduodenal hernia are clustering of small bowel loops, stretched, displaced and engorged mesenteric vessels with displacement of other bowel segments.

Many cases of laparoscopic repair of paraduodenal hernia have been reported, but most of them were of left side. Only three cases of laparoscopic repair of right paraduodenal hernia have been reported in the world's literature till date.[6–8] First case was reported by Antedomenico E. (2004) in a 24 year old woman associated with congenital malrotation.[6] Second reported case was a boy of 13 years diagnosed incidentally following a road traffic accident.[7] Patient details of third case were not present.[8] Our case was one of the youngest patient among the reported cases. She presented with features of obstruction but no strangulation and had the longest followup. There was no obvious malrotation. We reduced and repaired the hernia simply without severing lateral attachment of the colon.[7,8] The duration of hospital stay, first oral feed and recovery in our patient was similar to that of the other reported cases.

At least 50% of the patients with paraduodenal hernia develop partial or complete intestinal obstruction, therefore surgical correction is recommended to all.[6,8]

## CONCLUSION

Uematsu & Kitamura (1998) reported first the laparoscopic repair of paraduodenal hernia.[9] Several reports in the literature show that laparoscopic reduction and repair of the paraduodenal hernia offer the patient all the benefits of minimally invasive surgery and make the recovery faster so it can be recommended for all uncomplicated cases of paraduodenal hernia.[6,8,9]
